# Acceptability, Use, and Safety of a Mobile Phone App (BlueIce) for Young People Who Self-Harm: Qualitative Study of Service Users’ Experience

**DOI:** 10.2196/mental.8779

**Published:** 2018-02-23

**Authors:** Rebecca Grist, Joanna Porter, Paul Stallard

**Affiliations:** ^1^ Child and Adolescent Mental Health Group Department for Health University of Bath Bath United Kingdom; ^2^ Child and Family Mental Health Temple House Oxford Health National Health Service Foundation Trust Keynsham United Kingdom

**Keywords:** self-injurious behavior, mobile apps, adolescents, telemedicine, qualitative research, cognitive therapy, behavior therapy

## Abstract

**Background:**

Self-harm is common among adolescents and is associated with a number of negative psychosocial outcomes including a higher risk of suicide. Recent reviews highlight the lack of research into specific interventions for children and young people who self-harm. Developing innovative interventions that are coproduced with individuals with lived experience and that reduce self-harm are key challenges for self-harm prevention.

**Objective:**

The aim of this study was to explore the acceptability, use, and safety of BlueIce, a mobile phone app for young people who self-harm and who are attending child and adolescent mental health services (CAMHS).

**Methods:**

This study is part of a mixed methods phase 1 trial of BlueIce. Young people aged 12-17 years attending specialist CAMHS were recruited. Clinicians were invited to refer young people who were self-harming or who had a history of self-harm. On consent being obtained and baseline measures taken, participants used BlueIce as an adjunct to usual care for an initial familiarization period of 2 weeks. If after this time they wanted to continue, they used BlueIce for a further 10 weeks. Semistructured interviews were conducted at postfamiliarization (2 weeks after using BlueIce) and postuse (12 weeks after using BlueIce) to assess the acceptability, use, and safety of BlueIce. We undertook a qualitative analysis using a deductive approach, and then an inductive approach, to investigate common themes.

**Results:**

Postfamiliarization interviews were conducted with 40 participants. Of these, 37 participants elected to use BlueIce, with postuse interviews being conducted with 33 participants. Following 6 key themes emerged from the data: (1) appraisal of BlueIce, (2) usability of BlueIce, (3) safety, (4) benefits of BlueIce, (5) agency and control, and (6) BlueIce less helpful. The participants reported that BlueIce was accessible, easy to use, and convenient. Many highlighted the mood diary and mood lifter sections as particularly helpful in offering a way to track their moods and offering new strategies to manage their thoughts to self-harm. No adverse effects were reported. For those who did not find BlueIce helpful, issues around motivation to stop self-harming impeded their ability to use the app.

**Conclusions:**

BlueIce was judged to be a helpful and safe way of supporting adolescents to manage thoughts of self-harming. Adolescents reported numerous benefits of using BlueIce, and all would recommend the app to other young people who were struggling with self-harm. These preliminary findings are encouraging and provide initial support for the acceptability of BlueIce as a self-help intervention used in conjunction with the traditional face-to-face therapy.

## Introduction

### Background

Self-harm is a significant public health problem and is typically defined as an act of intentional self-injury or self-poisoning, irrespective of the type of motive or extent of suicidal intent [1]. Self-harm is common among adolescents and the prevalence is increasing. Community studies across different countries reliably report adolescents have a 13%-18% lifetime risk of self-harming [2-4]. In a UK-based community survey of adolescents aged 12-16 years, 1 in 4 reported self-harming thoughts and 1 in 6 had engaged in self-harming behavior over a 1-year period [5]. Repeated self-harming is common, with around half of adolescents who self-harm reporting doing so more than once [4-6]. Self-cutting appears to be the most common form of self-harm for adolescents in the community, whereas self-poisoning is the most common form seen in adolescents who present at hospital [4,7]. Only 1 in 8 adolescents who self-harm will present at hospital, the remainder of self-harm acts occur in private [4,7].

Self-harm is associated with a higher risk of mortality and suicide. Self-harm increases the likelihood that the person will eventually die by suicide [1]. Research indicates that of those who die by suicide, approximately 50% have a history of prior self-harm [8]. Although the risk of suicide in adolescents is low, it is the third leading cause of death for this age group worldwide [7,9]. Self-harm is also associated with a range of mental health problems such as depression, anxiety, substance misuse, eating disorders, and Attention Deficit Hyperactivity Disorder [1,7,10,11].

The National Institute of Health and Care Excellence (NICE) recommends offering 3 to 12 sessions of a psychological intervention that is specifically structured for people who self-harm, with the aim of reducing self-harm [1]. NICE also recommends the intervention contain either cognitive-behavioral, psychodynamic, or problem-solving elements [1]. Despite these recommendations, there is a paucity of research into specific interventions for children and adolescents who self-harm [11]. Evidence suggests that dialectical behavioral therapy (DBT) and cognitive behavioral therapy (CBT) warrant further research [11], and the key challenges for future research are the development and assessment of innovative interventions that are acceptable to young people, which reduce the risk of self-harm and enhance meaningful engagement with health services [7].

The last decade has seen a proliferation of mobile phone apps, and there are now more than 15,000 apps for health care with at least 29% designed for mental health [12]. The majority of adolescents now either own or have access to a mobile phone (96% of those aged 12-17 years) [13]. Mobile health (mHealth) therefore offers an accessible and potentially acceptable way to deliver and support mental health interventions for adolescents. A number of health organizations have developed policies for integrating mHealth and other digital technologies into health services, including child and adolescent mental health services (CAMHS) [14,15]. However, the quality and quantity of research evidence for the effectiveness of apps for mental health is scarce, particularly for adolescents [16].

It is imperative then that mobile phone apps for adolescents are subject to proper research evaluation [16] and ideally, codesigned with individuals with lived experience [11]. There is currently a paucity of research exploring the acceptability and safety of mobile apps for adolescents with mental health difficulties. A recent review of the research literature demonstrated that there are only a few feasibility studies exploring adolescent perspectives of mobile apps for mental health and only a minority of these included participants with lived experience of mental health difficulties [16]. To the knowledge of the authors, there are no mobile apps available for adolescents who self-harm that have been subject to published research evaluation.

### Objective

When young people self-harm, they are usually on their own, but most have access to a mobile phone. We developed and coproduced a mobile phone app (BlueIce) with young people with lived experience of self-harm. A series of meetings with young people who had lived experience of self-harm ensured coproduction and design of BlueIce [17]. The original idea for BlueIce was discussed with these young people. Further meetings with young people focused on app content, design, and presentation. A beta version of BlueIce was produced. This beta version was reviewed by the young people and a group of child mental health professionals who provided further recommendations. These recommendations were implemented in the second beta version that was reviewed again and positively endorsed by the young people. Full details of BlueIce development are reported in the study protocol [17].

BlueIce provides a personalized toolbox of strategies that can be accessed at any time and is intended to be used any time between face-to-face sessions. These strategies are based on theoretical approaches including DBT, CBT, mindfulness, and behavioral activation. BlueIce includes a mood diary, a menu of personalized mood lifting activities, and automatic routing through safety checks to delay or prevent self-harm. Mood lifting activities are designed to improve mood and include a personalized music and photo library, physical activities, mood changing activities, audio recordings of relaxation and mindfulness exercises, identification and challenging of negative thoughts, and distress tolerance activities. The young person is routed to emergency numbers if after using the mood lifter, the person reports that the thoughts of self-harming have not reduced. BlueIce is intended to be used alongside the traditional face-to-face interventions that adolescents receive from CAMHS.

The aim of BlueIce is to help young people manage their emotions and to prevent nonsuicidal self-injury (NSSI). Measurable outcomes of BlueIce include changes in self-harm and psychological functioning including depression and anxiety. Quantitative outcomes are reported elsewhere [18]. The aim of this study was to explore the acceptability, usability, and safety of BlueIce with young people aged 12-17 years who are self-harming and attending CAMHS. This study forms one part of a mixed methods phase 1 trial [17,18]. Here we report the qualitative data from participant interviews. We explored how young people used BlueIce, what they found positive and negative about the app, and whether they could use BlueIce when experiencing thoughts to self-harm.

## Methods

### Study Design

The study procedure consisted of an initial meeting (baseline), a familiarization period of 2 weeks and a second meeting (postfamiliarization), and then a further 10 weeks of app use and follow up meeting (postuse). We undertook semistructured interviews with young people at postfamiliarization and postuse. Interviews were conducted in person by the research team at locations convenient for the participant (generally either at home or at CAMHS). Interviews lasted around 40 min and were recorded and transcribed for analysis. Full study protocol is available [17]. The study was approved by the NHS South West—Exeter Research Ethics Committee (reference 16/SW/0018) and funded by the Health Foundation (2143 Oxford Health National Health Service [NHS] Foundation Trust).

### Setting and Sample

We conducted the study within CAMHS provided by Oxford Health NHS Foundation Trust. The Trust provides mental health care for children and young people in Buckinghamshire, Oxfordshire, Swindon, Wiltshire, and Bath and North-East Somerset.

Young people were eligible to take part if they were aged between 12 and 17 years and either currently self-harming or had a history of self-harm and felt that they would harm themselves again. All participants were required to be receiving face-to-face care from CAMHS for the duration of the study. Exclusion criteria included active suicidal ideation or planning, a diagnosis of psychosis or a significant learning disability, a current safeguarding investigation or having been subject to abuse in the last 6 months, or an inability to understand English.

### Recruitment and Consent

We initially provided study information to all clinical teams across Oxford Health NHS Trust via email. This was followed by meetings with clinical teams to demonstrate BlueIce and discuss any concerns or questions about the research. We conducted several other meetings with clinical teams following these initial meetings to maintain awareness of the project. All clinicians were given project information sheets and were asked to discuss the study with young people who they were working with and who met the inclusion criteria. If the young person indicated they would like to take part, the clinician passed their contact information and details of whether the young person had self-harmed in the last 4 weeks to the research team.

A member of the research team made contact and arranged to meet the young person and their carers (if they were younger than 16 years). At this initial meeting, the study was discussed and written consent was obtained from the young person. Parental consent was also obtained if the adolescent was younger than 16 years. At the initial meeting, the BlueIce app was downloaded onto the young person’s phone. If the young person either did not have a phone or owned an iPhone, they were given an Android phone with BlueIce preloaded. The young person personalized the mood lifter section of BlueIce with the researcher. Participants were recruited between May and November 2016.

### Role of Clinicians

Clinicians were responsible for identifying young people on their caseload who met the inclusion criteria and for providing them with project information. Consenting participants were provided with BlueIce, but they continued to attend face-to-face meetings with their clinician. As is usual practice, the clinician was responsible for reviewing risk and maintaining clinical responsibility for the young person in their care.

Clinicians had no role in setting up BlueIce with the participants and no role in providing technical assistance. There was no formal requirement for clinicians to discuss BlueIce with the young people in clinical sessions, and it was up to the young person if they wished to discuss the app with their clinician or not.

### Data Collection

Semistructured interviews were conducted to capture participant’s views broadly exploring the safety, acceptability, and usability of BlueIce. The postfamiliarization interviews incorporated the following aspects: (1) a general overview of how participants found using BlueIce, (2) what the participant liked and disliked about BlueIce, (3) what parts of BlueIce were particularly helpful and unhelpful, (4) any problems using BlueIce, (5) the degree to which participants thought BlueIce would help them prevent self-harm, and (6) the degree to which they thought BlueIce would make them feel like self-harming more. Interviews conducted at postuse included questions concerning the following: (1) if the participant had used BlueIce and reasons why or why not, (2) whether they used BlueIce when distressed, (3) if they used BlueIce to prevent self-harm and whether the app was helpful, (4) if they used BlueIce but it did not prevent an act of self-harm and why the app was not helpful, (5) what was helpful and unhelpful about BlueIce, (6) any problems or changes they would like to see, (7) the degree to which they found BlueIce to be easy or not easy and helpful or unhelpful, and (8) whether they would recommend BlueIce to other young people who self-harm.

Quantitative assessments were also completed at baseline, postfamiliarization, and postuse. These assessments included the Mood and Feelings Questionnaire (MFQ; assessing depression [19]), the Revised Child Anxiety and Depression Scale (RCADS; assessing anxiety [20]), and the Strengths and Difficulties Questionnaire (SDQ; assessing behaviors [21]). The results of which are available in the study by Stallard et al [18].

### Analysis

Each interview was audio-recorded, subsequently transcribed verbatim, and manually coded. We took a thematic approach to search across the data and identify themes following the 6 phases of thematic analysis outlined by Braun and Clark [22]. This included the following: (1) familiarizing ourselves with the data; individual transcripts were read and reread and notes of interest were taken, (2) generating initial codes; a list of initial codes was devised first from a deductive approach based on our research questions (safety, acceptability, and usability of BlueIce) then from an inductive approach to ensure that the main emerging ideas in the data were captured, (3) searching for themes; collecting candidate themes, sub themes, and data extracts in relation to each theme, (4) reviewing themes; systematically reading the collated data extracts for each theme, checking for consistency, and checking that our themes accurately represent the dataset, (5) defining and naming themes, and (6) producing the report. Nvivo 10 (QSR International, Australia) was used to conduct the analysis. At each stage of the process, RG (researcher) and PS (principal investigator) met to discuss codes and themes and resolve any discrepancies, to improve reliability.

## Results

### Description of Participants

Over the duration of the trial, 37 different clinicians from 8 teams referred 54 young people. Of the 54 referrals, 4 did not meet the inclusion criteria, we were unable to contact 3 referrals, 2 dropped out of CAMHS before they could consent, and 1 declined to participate. The remaining 44 completed baseline assessments. Participants were predominantly girls 90% (40/44) with an average age of 15.98 years (standard deviation, SD=1.37) with 68% (30/44) having self-harmed at least once in the 4 weeks before starting the trial. At baseline, using recommended cut-offs, 95% (42/44) scored 29 or more on the MFQ, suggesting probable depression. Using age- and gender-adjusted cut-offs on the RCADS, 84% (37/44) screened positive for one or more anxiety disorders, and 84% (37/44) scored above cut-offs on the SDQ for a probable emotional disorder. On the RCADS, 94% (16/17) of parents rated their child above the cut-off for depression, and 88% (16/18) scored their child above the cut-off on the SDQ for significant emotional problems.

Postfamiliarization interviews were conducted with 90% (40/44) of participants. Of these, 92% (37/40) elected to use BlueIce, with post use interviews being conducted with 82% (33/40) of participants.

### Summary of Themes

From these data, 6 key themes emerged: (1) appraisal of BlueIce, (2) usability of BlueIce, (3) safety (4) benefits of BlueIce, (5) agency and control, and (6) BlueIce less helpful. Table 1 details each theme including a definition and subthemes contained within.

#### Appraisal of BlueIce

Young people frequently reported that they found BlueIce to be helpful. The app was judged to provide a range of different techniques and strategies that offered a variety of options to manage self-harming urges. This accessibility and variety was valued by young people:

I’ve found it really helpful because I’ve tried using other apps and stuff but they only really cover like one aspect of what BlueIce offers…having app where there’s everything that you need like a little tool kit I think that’s really helpful

Participant number 142

An overwhelming majority of young people would recommend BlueIce to others who were self-harming. The 7 young people who reported that their self-harm did not reduce after using the app would still recommend BlueIce to others. They recognized that it could be beneficial for other people even though it did not directly benefit them.

Many young people also reported that BlueIce might benefit a wide range of people, not only those struggling with thoughts to self-harm:

My mum has quite a few mental illnesses and she had a look at the app and she actually really liked it as well so I don’t know if it would be available to adults as well?

Participant number 120

BlueIce was designed to be discreet and not draw attention to or expose the young person’s difficulties if someone else were to pick up their phone. This privacy was reported to be a significant benefit of the app:

It’s perfectly discreet and like it doesn’t have to be something that has to be hidden…it’s just, I like the design of it, I think it’s very well designed

Participant number 111

Young people also commented that they would like the ability to personalize BlueIce even more, and some noted the potential benefit of adding games into the app as another distraction technique:

Being able to personalise the colour, I thought that would be quite cool.

Participant number 113

#### Usability of BlueIce

Many young people who used BlueIce described the app as being easy to use, accessible, simple, and easy to navigate around. This ease of use was valued by participants as they did not want to add further stress to their situation:

It was really easy to get into and start using if you know what I mean, it was like once you knew how to use it, it was really easy.

Participant number 126

The young people who had BlueIce loaded on their own phone reported having the app on their device was convenient. This was because they had immediate access to the app and they could easily personalize the music and photo libraries from files already on their phone. Not being able to access BlueIce on their own device was a significant barrier to engagement for those participants who were provided an Android phone.

**Table 1:** Themes, theme definition, and subthemes derived from thematic analysis.

Theme	Definition	Subthemes
Appraisal of BlueIce	Indicators of BlueIce acceptability	Helpful Simple Provides a range of activities and sections Would recommend to others Benefit to people with other mental health problems More personalization Add games
Usability of BlueIce	Ease and patterns of BlueIce usage	Easy to use Accessible Convenient on own device Barrier if not on own device Situational barriers to use Finding wording confusing Frequency of app usage declining over the course of the study Attrition due to therapeutic gain High-frequency user
Safety and impact of BlueIce on self-harm	Indicators of BlueIce safety and purpose fulfillment	Concerns about mood diary Reassuring BlueIce not making self-harm worse BlueIce used when felt like self-harm BlueIce stopped self-harm BlueIce stopped more self-harm after initial episode Reduction in self-harm
Benefits of BlueIce	Reported cognitive, behavioral, and affective benefits of using BlueIce	Mood tracking and noticing patterns Reminder things get better Reminder you are able to cope Identifying benefits on mood Identifying triggers of negative moods BlueIce as a catalyst for difficult conversations New and personalized strategies Reminder that they have strategies Slow or reframe thinking Distraction Positive affective change
Agency and control	Perceived lack of ability to control self-harm urges and control emotion regulation	Differing willingness to receive help Urges to self-harm too overwhelming Feeling too overwhelmed to use BlueIce Urge to self-harm out of my control Hopelessness about helpfulness of interventions
BlueIce less helpful	Experiences of participants for whom BlueIce did not reduce self-harm	Not ready to stop self-harming Motivation to use BlueIce Personal crisis needing more CAMHS^a^ contact Ambivalence about helpfulness of BlueIce

^a^CAMHS: child and adolescent mental health services.

Situational barriers were also reported by young people, for example, being in a situation in which using BlueIce would have drawn attention to themselves or a situation in which they were unable to implement the strategies in the app (being at school, nighttime, parents being present):

It happened a few times because the worst place is at school. And I usually didn’t bring the phone to school or didn’t want to get it out halfway through lesson

Participant number 162

The type of therapy participants were receiving in their ongoing face-to-face treatment may have impacted their perception of BlueIce. Specifically, if the participants had encountered CBT, mindfulness, or DBT techniques in their therapeutic work, this appeared to make that part of the mood lifter more understandable and used more. Of all the participants, 1 participant noted that their face-to-face work was important to understand why they were doing CBT and mindfulness techniques and why they were useful. The wording in the CBT section (*thinking traps*) was reported to be slightly confusing by 3 participants, as they had not encountered CBT in their therapeutic work. This was a barrier to engagement with these sections, but other sections were still utilized.

For many participants, engagement with BlueIce tended to be higher at the beginning, with use declining over the duration of the study. Most young people reported that this was because their thoughts of self-harm had reduced and they needed to use the app less. There were also a minority of young people who continued to be frequent users of BlueIce, either because they still struggled with thoughts of self-harm or they particularly liked using the mood diary to track their mood:

I have used BlueIce every day. I used the mood checker every day and found it quite easy to use.

Participant number 144

#### Safety

No adverse effects of using BlueIce were reported. This means that no clinician withdrew any young person from the study due to an escalation of risk or the emergence of suicidal planning or attempt. Furthermore, no participants had reported calling the emergency numbers provided on BlueIce.

The possibility that BlueIce might have unintentional consequences and increase unpleasant feelings was also explored. There were 2 young people who reported concerns that negative feelings might be triggered by reading about their own negative feelings or events or seeing many sad faces on the mood diary. However, both these young people also reported benefits from reflecting on their mood diary entries.

Young people frequently described having BlueIce as reassuring and that it made them feel safer that there was a tool they could use for instant support:

It does make you feel safer in a way because you’ve got that choice of if I do need help then it’s right there…which I thought was really good

Participant number 154

Young people reported using BlueIce when they had thoughts of self-harming. For other young people, BlueIce was used to stop further self-harm after an initial episode:

I used to self-harm nearly every day without fail but since having the app I haven’t self-harmed since really, like I’ve had two slip ups but that’s it so it…it’s definitely helped me a lot

Participant number 115

#### Benefits of BlueIce

Young people reported many positive responses to the mood diary and mood lifter sections, particularly changes in their cognitions and behaviors. The ability to notice patterns in mood was a frequently reported benefit of the mood diary. Mood tracking resulted in various benefits, including remembering that they could have happier times and that they are able to cope with bad times, seeing the advantage of implementing the techniques in the mood lifer section on their mood, and recording negative events in the day that may have contributed to a negative mood and identifying ways to change. Several participants also reported that BlueIce was a catalyst for starting conversations with others about their feelings, or that BlueIce was a place where thoughts and feelings that were too difficult to share with others could be externalized:

I really liked the diary ‘cos…I dunno, ‘cos especially when I’m feeling down I’m like ‘OH I’m always so sad’ or ‘what is the point’ but actually if I look back to the diary I can see that there were days when I was happy.

Participant number 135

Participants also reported numerous benefits of using the mood lifter section. In particular, this section gave young people new strategies for managing their thoughts to self-harm and prompted users to think about their own strategies that were personal to them and their interests. This section was frequently referred to as a reminder that they had strategies to manage their distress other than self-harming and that they had people, normally friends, who they could talk to for support. Young people also reported how the techniques in the mood lifter section provided a distraction for them, helped them relax, or helped them slow down and reframe their thinking in the moments they had thoughts to self-harm. Although each participant liked different aspects, participants tried all sections of the mood lifter:

It just kind of stopped like the whole rush of it, so it slowed everything down, made you think about what was actually going on

Participant number 157

#### Agency and Control

A number of participants noted that there were times when they are open to receiving support to stop self-harming and other times when they were less willing to accept help and to stop the act of self-harm. A common report was that on some occasions, their urges to self-harm were too intense to stop and too intense to use BlueIce. Feeling overwhelmed and overpowered by their thoughts to self-harm left young people feeling that their self-harming was out of their control and a sense of hopelessness about anything else they could do to help them at that point in time:

I might just sort of feel a bit wilful, sort of, somewhat want to stop myself from self-harming but sometimes I just want to self-harm and that’s the end of it really

Participant number 120

I think a couple of times when the urges were just really high and I kind of knew in the back of my mind that nothing was going to help really

Participant number 120

#### BlueIce Less Helpful

A subgroup of 7 adolescents did not find BlueIce helpful in reducing their self-harm. These young people tended to report an ambivalence about the helpfulness of BlueIce and that they rarely used it. A common thread among these young people was a sense that they were not ready to give up self-harming and they found it difficult to get motivated to use BlueIce:

I guess I was hesitant about trying it and I was also scared that it would work because…well…I don’t know, that means I could get better, but I don’t particularly want to get better

Participant number 117

Furthermore, at least 4 of the young people experienced personal crises during this study, which impacted their ability to use BlueIce. This generally involved an increased need for more face-to-face contact with CAMHS, which took precedence over using BlueIce:

I was more in contact with the crisis team and more involved with like CAMHs like more than once a week so I was kind of getting more things to do than just use the BlueIce app

Participant number 137

## Discussion

### Principal Findings and Comparison With Prior Work

The aim of this study was to explore the acceptability, use, and safety of BlueIce, a mobile phone app for young people who self-harm and attend CAMHS. Themes that transpired from our analysis suggested that BlueIce promoted positive changes for a number of adolescents, including helping to slow down or reframe their thinking, distraction from thoughts of self-harming, and identifying triggers of negative moods. Overall, BlueIce was deemed to be helpful, easy to use, and safe.

### Acceptability

The majority of adolescents reported that BlueIce was helpful for them: in particular, having a range of techniques available in one app, techniques that kept them distracted and their hands busy, having an outlet for their emotions, and quick access to emergency numbers were deemed most helpful. All participants would recommend BlueIce to others, with a number of adolescents spontaneously suggesting that BlueIce would be helpful to other mental health populations, besides young people who self-harm. These results are encouraging as perceived helpfulness of a mental health intervention is considered a key indicator of acceptability [23] and one of the most important criterion for choosing to use a mental health intervention [24].

BlueIce was designed to be private, discrete, and confidential to use. BlueIce is password protected, a level of privacy valued by young people. The app was therefore discrete on their phones; for example, if one of their friends used their phone, it would not be obvious that a mental health app was installed. Previous research has suggested that concerns around privacy and discretion are important factors to consider in a mental health app design [16,25,26]. This is deemed especially important for adolescents because of a perceived stigma around accessing mental health services [27]. Our study highlights that these factors are also important for young people experiencing mental health problems and who are self-harming. It is advisable, therefore, that mental health apps be developed with privacy and discretion in mind.

Previous research has indicated that young people want the ability to personalize mental health apps [25]. BlueIce includes a level of personalization, such as the ability to add activities personalized to the interests of the adolescent and the ability to add pictures and music from their personal device. These elements of personalization were valued, although many adolescents would like to personalize the app even further by adding different colors to the app. Similarly, many participants stated that adding a game to BlueIce would be beneficial [28] as another distraction technique. There is a potential mismatch here between the adolescents’ expectations and what could feasibly be produced within the resources available. We did, however, implement changes to BlueIce based on the feedback received. These changes included adding more options to the mood checker such as *OK* instead of *so-so* and *other*, which allows users to put in their own words about how they are feeling. Colors were added to each option on the mood wheel ranging from red (really sad) to green (really happy). As participants tended to report that they would prefer to text their contacts from BlueIce rather than call them, this option was also added.

### Usability

BlueIce was consistently judged to be simple and easy to use. Adolescents reported that the app was accessible, easy to navigate, and was not burdensome. Adolescents also reported that they used BlueIce for its intended purpose: to manage their thoughts of self-harming. Ease of use is particularly important for facilitating engagement with e-mental health interventions [29], and adolescents consider ease of use as an important design requirement of mental health apps [25]. Our study adds emphasis to previous design recommendations that mental health apps for adolescents should be easy to use and require as little training as possible [30].

Participants reported the mood diary and mood lifter sections as parts of the app they particularly liked and used. Tracking and recognizing patterns in moods and identifying triggers for negative emotions were deemed to be the major benefits of using the mood diary regularly. Young people who self-reported high and continued engagement with BlueIce noted the mood diary was a major motivator for this. Young people reported that self-monitoring also helped them to cope with periods of time when they felt less positive, because they could see that they were able to have happier days and the periods of negative emotions do pass. Self-monitoring also highlighted to adolescents that the mood lifting activities were having a positive impact on their mood, and therefore, they could identify the therapeutic benefits of using BlueIce. These findings suggest some participants were using self-monitoring to track and reflect on their emotions and develop a level of awareness about what may influence positive or negative moods. This development of emotional self-awareness has been demonstrated as a benefit of app-based self-monitoring in previous work with adolescents [31,32]. Our findings, therefore, correspond with previous research, which indicates that mood self-monitoring is reported to be one of the most liked and engaged with sections of mental health apps [31] and can potentially produce higher compliance with self-monitoring than the traditional paper-based methods [26,33].

Self-reported use of BlueIce tended to be higher at the start of the study, with usage commonly dropping over time. This usage pattern corresponds with those evident in previous work [34,35]. For example, in a feasibility study of an app for adolescent anxiety (SmartCAT; N=9, aged 9-14 years), high utilization was evident during week 1, but leveled off over time and halved by week 7 [34]. Similarly, in a feasibility trial of Mobiletype, a mood self-monitoring app for young people [35], participants (N *=* 47, aged 14-24 years) completed 91% (47/51) of the entries every day in week 1, dropping to 58% (17/29) in week 4. Participants in this study used BlueIce over a duration of 12 weeks—much longer than the lengths of previous studies [34,35]; therefore, an amount of attrition is to be expected.

Attrition from eHealth interventions is a common phenomenon [36], but the causes are not well understood. Of those who gave reasons for discontinuing to use BlueIce, some identified positive reasons, for example, that they had not needed to use it so much. This corresponds with a general improvement on mental health outcomes at postintervention [18] and number of reports from participants that they only tended to use BlueIce when they were feeling low.

Participants did also report certain barriers to engagement with BlueIce. Many participants reported that not having BlueIce on their own phone meant they forgot to use the app or felt uncomfortable using it in public. This has now been addressed and BlueIce is available on both iPhone and Android platforms. Participants also felt that there were situations in which it was difficult for them to use BlueIce, such as being in school or being around their family. In these instances, they could not use the strategies in the mood lifter section to regulate their emotions. These practical barriers are more difficult to overcome, as they cannot be mitigated by the app design. Discussing these barriers with CAMHS therapists and identifying solutions would be beneficial in these circumstances.

For the 7 young people who did not show any improvement in their self-harm during the study, a number of issues were highlighted. These included finding the app repetitive, not finding it helpful, experiencing annoyance when asked questions about how they were feeling, and 1 participant reported preferring to speak to someone face-to-face. Most of these participants reported not being ready to stop self-harming and therefore experienced a lack of motivation to use BlueIce. These young people tended to acknowledge that BlueIce would be helpful if they had the necessary motivation to use it, and some indicated they had little faith that any interventions could help them overcome their difficulties. The need to be committed to change and lack of motivation have been identified as barriers to mHealth engagement in both adults [37-39] and adolescents [40]. Mental health practitioners should, therefore, consider service user motivation and readiness to change when recommending mHealth apps as an adjunct to face-to-face care. Making mHealth apps intrinsically more engaging by design could be a way to increase longer-term engagement and motivation. One promising avenue is the use of serious gaming, gamification principles, telepresence, and persuasive technology [28]. How these principles may be incorporated into BlueIce is for further research to address.

### Safety

mHealth researchers have highlighted that mhealth apps can pose risks to patient safety and that steps should be taken to mitigate these risks [41,42]. Unfortunately, reporting of risks, adverse events, and safety of mental health apps is sparse in the current literature [16]. Given the nature of self-harm and associated comorbid difficulties, it was deemed critical to explore with young people whether BlueIce worked as intended, caused any unintentional adverse effects, or whether any other safety concerns arose during the study. Young people who used BlueIce reported that they used the app as intended, to help them manage their thoughts of self-harming. Some participants reported that BlueIce helped them not to act on their thoughts to self-harm. Others reported instances where BlueIce did not prevent an initial act of self-harm, but prevented them from continuing to harm themselves in that instance. Participants tended to acknowledge that BlueIce was helpful but would not be able to prevent every instance of self-harm. Participants explained that in these instances, they felt their emotions were too overwhelming and some reported feeling like nothing would have helped them in that moment. BlueIce is intended to be used as an adjunct to face-to-face meetings. This allows the young person’s progress to be regularly reviewed and additional interventions provided to increase motivation and to develop protective skills.

Although 2 participants did report initial concerns that seeing their mood diary full of negative days might not help them, they did reflect further that overall the benefits of self-monitoring would outweigh these concerns. No participants believed BlueIce would increase their thoughts of self-harming, and no adverse events were reported. Many of the participants stated they felt reassured to have the app when they needed it, and most participants wanted to keep BlueIce at the end of the study. Overall, our results provide preliminary support for the safety of BlueIce as an adjunct to face-to-face therapy.

### Limitations

Although our results are encouraging, our study does have some limitations. First, we report a self-selected case series of young people who actively elected to try BlueIce. Our group may therefore have had more positive attitudes toward using an mHealth intervention. Second, due to the interviews being conducted by research assistants, there is a possibility that participants reporting may be subject to bias. It should be noted, however, that the research assistants were not involved in the initial development of BlueIce and went to great lengths to reassure participants that negative feedback would be helpful to make changes to the app. Third, 37 participants chose to use BlueIce after the 2-week familiarization period, with postuse interviews being conducted with 33. We could not gain contact with the 4 participants who dropped out the study, and therefore, we do not have their feedback on why they discontinued the study, be it because of the research process, their experiences of using BlueIce, or other factors. Similarly, several participants did not find BlueIce helpful in reducing their self-harm. We must be mindful that BlueIce is a mobile phone app and that there is a limit to which it can stop all acts of self-harm. Although many participants in this category reported feeling that nothing (ie, no intervention) would have helped them in that moment, the limitations of digital support should be acknowledged.

We also acknowledge that some of the questions in the postuse interview maybe subject to recall bias. For example, asking participants what factors influenced them to use or not use BlueIce in a moment of distress when they were struggling with thoughts of self-harm. This may be overcome in a subsequent phase 2 trial by the use of ecological momentary assessment methods and sampling as close to real time as possible.

Finally, BlueIce was provided in addition to usual face-to-face intervention. As such, there may be possible synergic effects between the face-to-face intervention and the usability, acceptability, and safety of the app. A randomized trial comparing treatment as usual with and without BlueIce is required to explore this further and gain better insight into the acceptability, use, and safety of BlueIce, beyond this exploratory, uncontrolled study.

### Conclusions

To the knowledge of the authors, this is the first reported qualitative study detailing the experience of adolescents using an app specifically developed with young people for young people who self-harm. Interviews with adolescents who had used BlueIce demonstrated that it was considered an acceptable, easy, and safe app to use that helped young people manage their thoughts of self-harm. Young people reported a number of advantages of using BlueIce, in particular being able to track their mood and access techniques to help distract them from their thoughts of self-harm. Given the high acceptability of BlueIce, this study demonstrates the value of meaningful involvement of adolescents with lived experience in the design, production, and implementation of mHealth interventions. This study provides initial support for the acceptability, usability, and safety of BlueIce as an mHealth intervention for preventing self-harm used in conjunction with the traditional face-to-face therapy. A subsequent phase 2 randomized controlled trial is now warranted to demonstrate the effectiveness of BlueIce in reducing adolescent self-harm.

## Abbreviations

CBT: cognitive behavioral therapy
CAMHS: child and adolescent mental health services
DBT: dialectical behavioral therapy
mHealth: mobile health
MFQ: Mood and Feelings Questionnaire
NICE: National Institute of Health and Care Excellence
NSSI: nonsuicidal self-injury
RCADS: Revised Child Anxiety and Depression Scale
SDQ: Strengths and Difficulties Questionnaire

## References

[1] National Institute for Health Care Excellence (2011). NICE.

[2] Mars B, Heron J, Crane C, Hawton K, Lewis G, Macleod J, Tilling K, Gunnell D (2014). Clinical and social outcomes of adolescent self harm: population based birth cohort study. Br Med J.

[3] Kidger J, Heron J, Lewis G, Evans J, Gunnell D (2012). Adolescent self-harm and suicidal thoughts in the ALSPAC cohort: a self-report survey in England. BMC Psychiatry.

[4] Madge N, Hewitt A, Hawton K, de Wilde EJ, Corcoran P, Fekete S, van Heeringen K, De Leo D, Ystgaard M (2008). Deliberate self-harm within an international community sample of young people: comparative findings from the Child & Adolescent Self-harm in Europe (CASE) Study. J Child Psychol Psychiatry.

[5] Stallard P, Spears M, Montgomery AA, Phillips R, Sayal K (2013). Self-harm in young adolescents (12-16 years): onset and short-term continuation in a community sample. BMC Psychiatry.

[6] Hawton K, Bergen H, Kapur N, Cooper J, Steeg S, Ness J, Waters K (2012). Repetition of self-harm and suicide following self-harm in children and adolescents: findings from the Multicentre Study of Self-harm in England. J Child Psychol Psychiatry.

[7] Hawton K, Saunders KE, O'Connor RC (2012). Self-harm and suicide in adolescents. Lancet.

[8] Hawton K, Bergen H, Kapur N, Cooper J, Steeg S, Ness J, Waters K (2012). Repetition of self-harm and suicide following self-harm in children and adolescents: findings from the Multicentre Study of Self-harm in England. J Child Psychol Psychiatry.

[9] Patton GC, Coffey C, Sawyer SM, Viner RM, Haller DM, Bose K, Vos T, Ferguson J, Mathers CD (2009). Global patterns of mortality in young people: a systematic analysis of population health data. Lancet.

[10] Hawton K, Saunders K, Topiwala A, Haw C (2013). Psychiatric disorders in patients presenting to hospital following self-harm: a systematic review. J Affect Disord.

[11] Hawton K, Witt KG, Taylor ST, Arensman E, Gunnell D, Townsend E, van HK, Hazell P (2015). Interventions for self-harm in children and adolescents. Cochrane Database Syst Rev.

[12] Anthes E (2016). Mental health: there's an app for that. Nature.

[13] (2013). eMarketer.

[14] The National Institute for Health Research Horizon Scanning Research Intelligence Centre (2015). io.nihr.

[15] East ML, Havard BC (2015). Mental health mobile apps: from infusion to diffusion in the mental health social system. JMIR Ment Health.

[16] Grist R, Porter J, Stallard P (2017). Mental health mobile apps for preadolescents and adolescents: a systematic review. J Med Internet Res.

[17] Stallard P, Porter J, Grist R (2016). Safety, acceptability, and use of a smartphone app, BlueIce, for young people who self-harm: protocol for an open phase I trial. JMIR Res Protoc.

[18] Stallard P, Porter J, Grist R (2018). A smartphone app (BlueIce) for young people who self-harm: open phase 1 pre-post trial. JMIR Mhealth Uhealth.

[19] Wood A, Kroll L, Moore A, Harrington R (1995). Properties of the mood and feelings questionnaire in adolescent psychiatric outpatients: a research note. J Child Psychol Psychiatry.

[20] Chorpita BF, Moffitt CE, Gray J (2005). Psychometric properties of the Revised Child Anxiety and Depression Scale in a clinical sample. Behav Res Ther.

[21] Goodman R (1997). The Strengths and Difficulties Questionnaire: a research note. J Child Psychol Psychiatry.

[22] Braun V, Clarke V (2006). Using thematic analysis in psychology. Qual Res Psychol.

[23] Apolinário-Hagen J, Kemper J, Stürmer C (2017). Public acceptability of e-mental health treatment services for psychological problems: a scoping review. JMIR Ment Health.

[24] Musiat P, Goldstone P, Tarrier N (2014). Understanding the acceptability of e-mental health--attitudes and expectations towards computerised self-help treatments for mental health problems. BMC Psychiatry.

[25] Kenny R, Dooley B, Fitzgerald A (2016). Developing mental health mobile apps: exploring adolescents' perspectives. Health Informatics J.

[26] Matthews M, Doherty G (2011). In the mood: Engaging teenagers in psychotherapy using mobile phones. Proceedings of the SIGCHI Conference on Human Factors in Computing Systems.

[27] Bulanda JJ, Bruhn C, Byro-Johnson T, Zentmyer M (2014). Addressing mental health stigma among young adolescents: evaluation of a youth-led approach. Health Soc Work.

[28] Fleming TM, De Beurs D, Khazaal Y, Gaggioli A, Riva G, Botella C, Baños RM, Aschieri F, Bavin LM, Kleiboer A, Merry S, Lau HM, Riper H (2016). Maximizing the impact of e-therapy and serious gaming: time for a paradigm shift. Front Psychiatry.

[29] Povey J, Mills PP, Dingwall KM, Lowell A, Singer J, Rotumah D, Bennett-Levy J, Nagel T (2016). Acceptability of mental health apps for Aboriginal and Torres Strait islander Australians: a qualitative study. J Med Internet Res.

[30] Matthews M, Doherty G, Coyle D, Sharry J, Lumsden J (2008). Designing mobile applications to support mental health interventions. Handbook of research on user interface design and evaluation for mobile technology.

[31] Kenny R, Dooley B, Fitzgerald A (2015). Feasibility of “CopeSmart”: a telemental health app for adolescents. JMIR Ment Health.

[32] Reid SC, Kauer SD, Dudgeon P, Sanci LA, Shrier LA, Patton GC (2009). A mobile phone program to track young people's experiences of mood, stress and coping. Development and testing of the mobiletype program. Soc Psychiatry Psychiatr Epidemiol.

[33] Matthews M, Doherty G, Sharry J, Fitzpatrick C (2008). Mobile phone mood charting for adolescents. Brit J Guid Couns.

[34] Pramana G, Parmanto B, Kendall PC, Silk JS (2014). The SmartCAT: an m-health platform for ecological momentary intervention in child anxiety treatment. Telemed J E Health.

[35] Reid SC, Kauer SD, Khor AS, Hearps SJ, Sanci LA, Kennedy AD, Patton GC (2012). Using a mobile phone application in youth mental health - an evaluation study. Aust Fam Physician.

[36] Eysenbach G (2005). The law of attrition. J Med Internet Res.

[37] Peng W, Kanthawala S, Yuan S, Hussain SA (2016). A qualitative study of user perceptions of mobile health apps. BMC Public Health.

[38] Ly K, Janni E, Wrede R, Sedem M, Donker T, Carlbring P, Andersson G (2015). Experiences of a guided smartphone-based behavioral activation therapy for depression: a qualitative study. Internet Interv.

[39] Svensson A, Magnusson M, Larsson C (2016). Overcoming barriers: adolescents' experiences using a mobile phone dietary assessment app. JMIR Mhealth Uhealth.

[40] Lewis TL, Wyatt JC (2014). mHealth and mobile medical apps: a framework to assess risk and promote safer use. J Med Internet Res.

[41] Naeem F, Gire N, Xiang S, Yang M, Syed Y, Shokraneh F, Adams C, Farooq S (2016). Reporting and understanding the safety and adverse effect profile of mobile apps for psychosocial interventions: an update. World J Psychiatry.

[42] Whittaker R, Merry S, Stasiak K, McDowell H, Doherty I, Shepherd M, Dorey E, Parag V, Ameratunga S, Rodgers A (2012). MEMO--a mobile phone depression prevention intervention for adolescents: development process and postprogram findings on acceptability from a randomized controlled trial. J Med Internet Res.

